# LncRNA-ATB promotes trastuzumab resistance and invasion-metastasis cascade in breast cancer

**DOI:** 10.18632/oncotarget.3457

**Published:** 2015-03-23

**Authors:** Sheng-Jia Shi, Li-Juan Wang, Bo Yu, Yun-Hui Li, Yong Jin, Xiao-Zhong Bai

**Affiliations:** ^1^ Department of Administration and Department of Aristogenesis, No. 202 Hospital of PLA, No. 5, Shenyang, 110003, Liaoning Province, P.R. China; ^2^ Department of Oncology, the First Affiliated Hospital of Medical College of Xi'an Jiaotong University, Xi'an, 710061, Shaanxi Province, P.R. China

**Keywords:** lnc-ATB, trastuzumab resistance, EMT, TGF-β, Breast cancer

## Abstract

Trastuzumab resistance is leading cause of mortality in HER2-positive breast cancers, and the role of TGF-β-induced epithelial-mesenchymal transition (EMT) in trastuzumab resistance is well established, but the involvement of lncRNAs in trastuzumab resistance is still unknown. Here, we generated trastuzumab-resistant breast cancer cells with increased invasiveness compared with parental cells, and observed robust epithelial–mesenchymal transition (EMT) and consistently elevated TGF-β signaling in these cells. We identified long noncoding RNA activated by TGF-β (lnc-ATB) was the most remarkably upregulated lncRNA in TR SKBR-3 cells and the tissues of TR breast cancer patients. We found that lnc-ATB could promote trastuzumab resistance and invasion-metastasis cascade in breast cancer by competitively biding miR-200c, up-regulating ZEB1 and ZNF-217, and then inducing EMT. In addition, we also found that the high level of lnc-ATB was correlated with trastuzumab resistance of breast cancer patients. Thus, these findings suggest that lncRNA-ATB, a mediator of TGF-β signaling, could predispose breast cancer patients to EMT and trastuzumab resistance.

## INTRODUCTION

Breast cancer is the most prevalent malignance in women with 232670 newly estimated diagnosed cases and 40000 estimated deaths in the United States in 2014 [1]. The leading causes of these deaths are attributed to distal metastasis and resistance to currently available therapeutics [2]. Erb2/HER2 is amplified and overexpressed in 25%–30% of human breast cancers [3]. HER2-positive breast cancer has the second-poorest prognosis among breast cancer subtypes and is correlated with reduced disease-free and overall survival time [4–5]. Trastuzumab (Herceptin; Genetech, San Francisco, CA, USA) is designed to target the extra-cellular domain of human epidermal growth factor receptor 2 (HER2) and block its function, and is currently used in patients with early stage and metastatic HER2-positive breast and gastric cancers. Even though trastuzumab-based combination treatment with chemotherapy has significantly improved outcome in breast cancer patients and has paved the way for the era of targeted therapy in breast cancer treatment, the median duration of response is modest [6–7]. This is due to either primary or secondary mechanisms of resistance during the course of treatment [8–9]. More importantly, only less than 35% of patients with HER2-positive breast cancer initially respond to trastuzumab [10–11]. Though a number of critical studies have been conducted to identify the underlying mechanisms that account for trastuzumab resistance, little is known regarding the biological role of long non-coding RNA (lncRNA) in the process of trastuzumab resistance.

The multifunctional cytokine transforming growth factor β (TGF-β) orchestrates an intricate signaling network to modulate carcinogenesis and progression [12]. It has been confirmed that TGF-β signaling could promote cancer progression and confer resistance to various therapeutics through enhancing proliferation, migration, and invasion, in part by its ability to induce epithelial-mesenchymal transition (EMT) [13–15]. EMT has been proven to correlate closely with cancer progression by facilitating invasion of cancer cells [16–17]. In breast cancers, the functional synergy between TGF-β and HER2 has been characterized. It has been demonstrated that TGF-β cooperated with HER2 at various levels, including transcriptional regulation of the Smad target genes and pathways; activation of the PI3K/Akt pathway in a Smad-independent manner; modification of the tumor microenvironment by inducing the secretion of TGF-β, Erb2 ligands, and angiogenic mediators [18]. Given the established role of EMT in breast cancer progression [19], deciphering the biological function of TGF-β signaling in trastuzumab resistance and metastasis of HER2 positive breast cancer will provide novel insights into molecular mechanism underlying multifaceted malignant phenotypes of breast cancer.

LncRNAs are a class of transcripts longer than 200 nucleotides with extremely limited protein coding potential. Recently, many studies have demonstrated that lncRNAs had multiple functions in a wide range of biological process, such as proliferation, apoptosis, cell migration, and cell invasion [20–21]. Although lncRNAs have been reported to modulate tumor metastasis by mediating the prometastatic role of TGF-β and regulating EMT [22], and several lncRNA transcripts were involved in the biology of carcinogenesis in breast cancer [23–24], the biological roles of lncRNAs in the process of trastuzumab resistance in breast cancer are not well studied.

In the current study, we screened for candidate lncRNAs responsible for the maglinant phenotype of trastuzumab resistant (TR) SKBR-3. We identified long noncoding RNA activated by TGF-β (lnc-ATB) was the most remarkably up-regulated lncRNA in TR SKBR-3 cells and the tissues of TR breast cancer patients. We then focus on the role of lnc-ATB in trastuzumab resistance and invastion-metastasis cascade of breast cancer. We found lnc-ATB could promote trastuzumab resistance and invasion-metastasis cascade in breast cancer by competitively biding miR-200c, up-regulating ZEB1 and ZNF-217, and then inducing EMT.

## RESULT

### Induction and identification the phenotype of trastuzumab resistant SKBR-3 model

**Figure 1:**
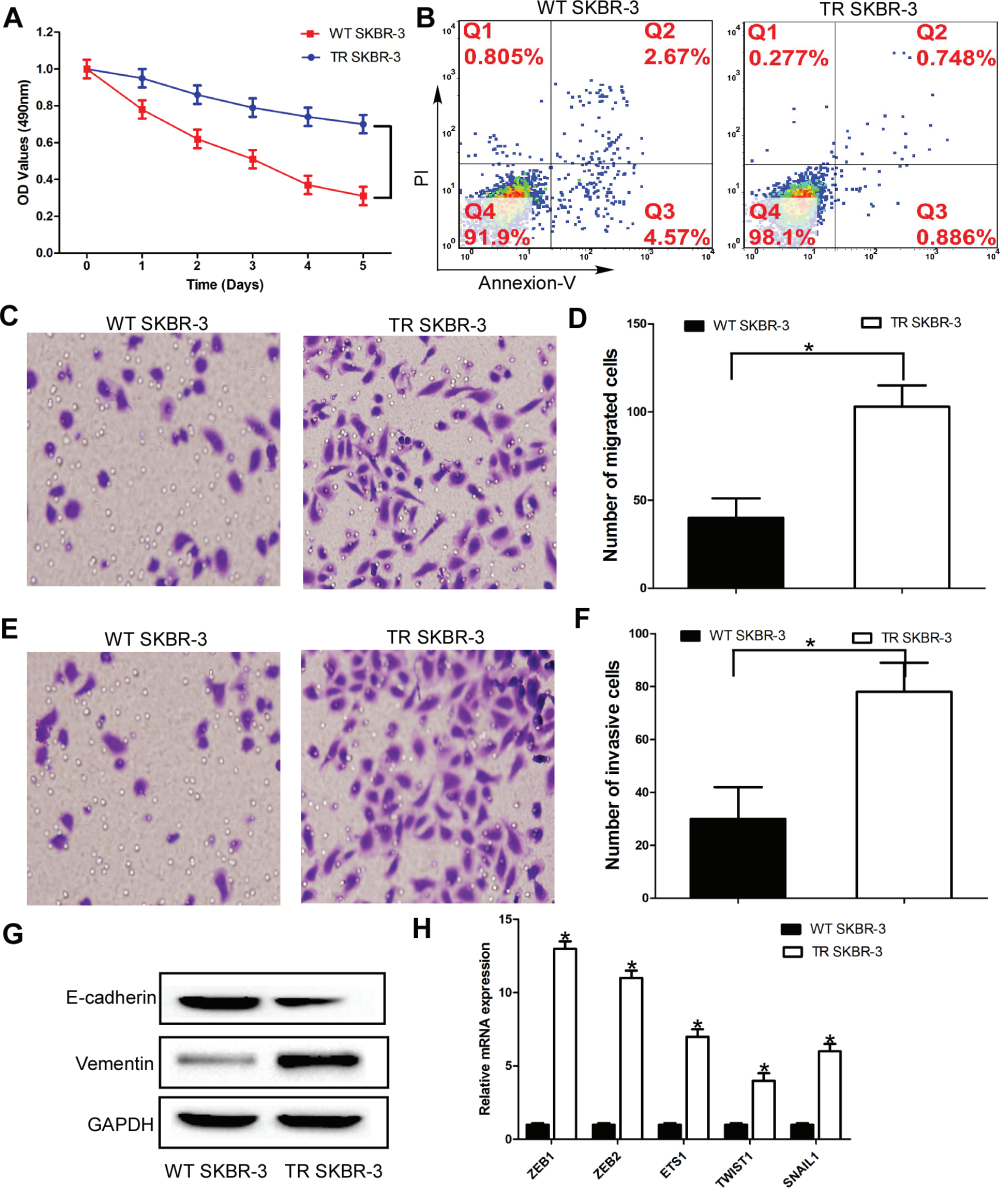
Elevated invasiveness and EMT of trastuzumab-resistant breast cancer cells **(A)** TR and WT SKBR-3 cells were exposed to trastuzumab for 24 h and subjected to MTT assays; **(B)** Apoptosis rate of TR and WT SKBR-3 cells; **(C)** and **(D)** TR and WT SKBR-3 cells subjected to migration assays; **(E)** and **(F)** TR and WT SKBR-3 cells subjected to invasion assays; **(G)** Expression of E-cadherin and vementin in TR and WT SKBR-3 cells; **(H)** Expression of EMT markers in TR and WT SKBR-3 cells; Data are expressed as means ± SD. Two-tailed Student's *t*-test was used to analyze the significant differences. *P < 0.05.

In order to develop an *in vitro* SKBR-3 model of acquired trastuzumab resistance, SKBR-3 which is HER-2 overexpressed breast cancer cell line was continuously exposed to 5 μg/ml trastuzumab for 6 months until cells had acquired trastuzumab resistance [25]. Consistent with previous studies characterizing trastuzumab resistant breast cancer cells (TR), we observed the growth inhibition of TR SKBR-3 cells was significantly lower than WT SKBR-3 cells under the trastuzumab medium explosion (Figure 1A) [26].

Then, we investigated the difference of malignant phenotype between TR SKBR-3 cells and WT SKBR-3 cells. Compared with WT SKBR-3 cells, the apoptosis rate of TR SKBR-3 cells was significantly decreased (Figure 1B). Moreover, the migration and invasion capacity of TR SKBR-3 cells was significantly increased than WT SKBR-3 cells (Figure 1C–1F). Consistent with the morphological changes of EMT, the epithelial maker E-cadherin was significantly down-regulated in TR SKBR-3 cells; meanwhile, the mesenchymal marker Vimentin was significantly up-regulated in SKBR-3 cells (Figure 1G). A cluster of EMT markers, including ZEB1, ZEB2, ETS1, TWIST1, SNAIL1, was significantly increased in TR SKBR-3 cells (Figure 1H). These results indicated the acquisition of trastuzumab significantly enhanced the migration and invasion capacity of breast cancer cells, and the underlying mechanism of this phenomenon is EMT of breast cancer cells.

### Lnc-ATB is up-regulated in TR breast cancer tissues and TR SKBR-3 cells

**Figure 2:**
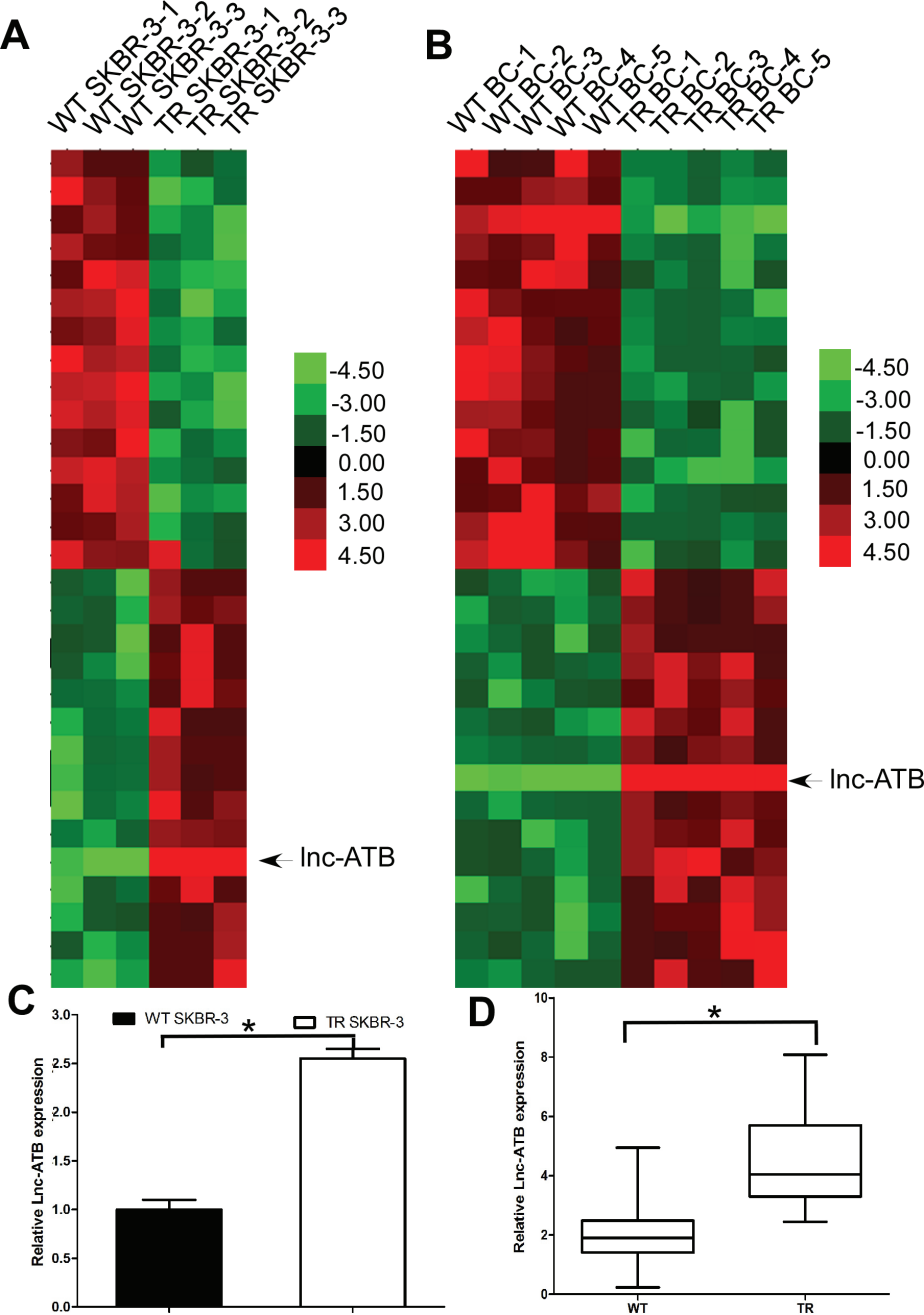
Lnc-ATB is up-regulated in TR breast cancer tissues and TR SKBR-3 cells **(A)** Expression profile of lncRNAs in TR and WT SKBR-3 cells; **(B)** Expression profile of lncRNAs in tissues of TR and WT breast cancer patients; **(C)** Expression of lnc-ATB in TR and WT SKBR-3 cells; **(D)** Expression of lnc-ATB in tissues of TR and WT breast cancer patients. Data are expressed as means ± SD. Two-tailed Student's *t*-test was used to analyze the significant differences. *P < 0.05.

LncRNAs has been proven to play critical role in mediating the prometastatic role of TGF-β and regulating EMT. We used microarray analysis to compare the lncRNA expression level between the TR SKBR-3 and WT SKBR-3, we found 572 up-regulated and 321 down-regulated lncRNAs in TR SKBR-3 cells. Then, we used microarray analysis to compare the lncRNA expression level between the tissues of 5 WT breast cancer patients and 5 TR breast cancer patients, we found 421 up-regulated and 245 down-regulated lncRNAs in TR breast cancer patients. Among the 30 lncRNAs with the most significant fold change in tissues of TR breast cancer patients or TR SKBR-3 cells, lnc-ATB was found up-regulated in both TR SKBR-3 cells and tissues of TR breast cancer patients (Figure 2A and 2B). Given the established role of lnc-ATB in invasion and EMT, we proposed lncATB should account for the aforementioned changes of malignant phenotypes between WT SKBR-3 and TR SKBR-3 cells. We then further confirmed the significant up-regulation of lnc-ATB in TR SKBR-3 cells (Figure 2C) and tissues of 50 TR breast cancer patients (Figure 2D).

### Up-regulation of lnc-ATB account for the trastuzumab resistance and high invasiveness of TR SKBR-3 cells

**Figure 3:**
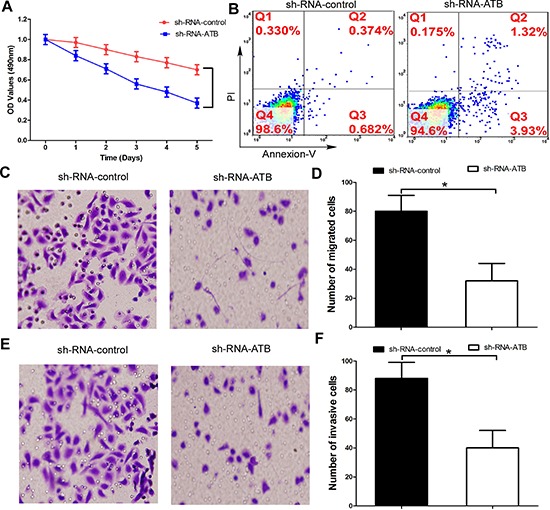
Up-regulation of lnc-ATB account for the trastuzumab resistance and high invasiveness of TR SKBR-3 cells **(A)** TR SKBR-3 cells transfected with lnc-ATB or control subjected to MTT assays; **(B)** Apoptosis rate of TR SKBR-3 cells transfected with lnc-ATB or control; **(C)** and **(D)** TR SKBR-3 cells transfected with lnc-ATB or control subjected to migration assay; **(E)** and **(F)** TR SKBR-3 cells transfected with lnc-ATB or control subjected to invasion assay. Data are expressed as means ± SD. Two-tailed Student's *t*-test was used to analyze the significant differences. *P < 0.05.

In order to further confirm whether lnc-ATB promotes trastuzumab resistance through regulating EMT, we performed MTT assays to investigate the effects of lnc-ATB on malignant phenotypes of TR SKBR-3 cells. Down-regulation of lnc-ATB significantly increased the growth inhibition of TR SKBR-3 cells under the under the trastuzumab medium explosion (Figure 3A). Furthermore, down-regulation of lnc-ATB also significantly increased the apoptosis rate of TR SKBR-3 cells (Figure 3B). More importantly, compared with shRNA-control, TR SKBR-3 cells transfected with shRNA-ATB significantly decreased the capacity of migration and invasion (Figure 3C–3F). Taken together, these results indicated lnc-ATB could promote trastuzumab resistance through increasing the invasiveness and EMT of breast cancer cells.

### miR-200c is down-regulated and inverse correlated with lnc-ATB in TR breast cancer tissues and TR SKBR-3 cells

**Figure 4:**
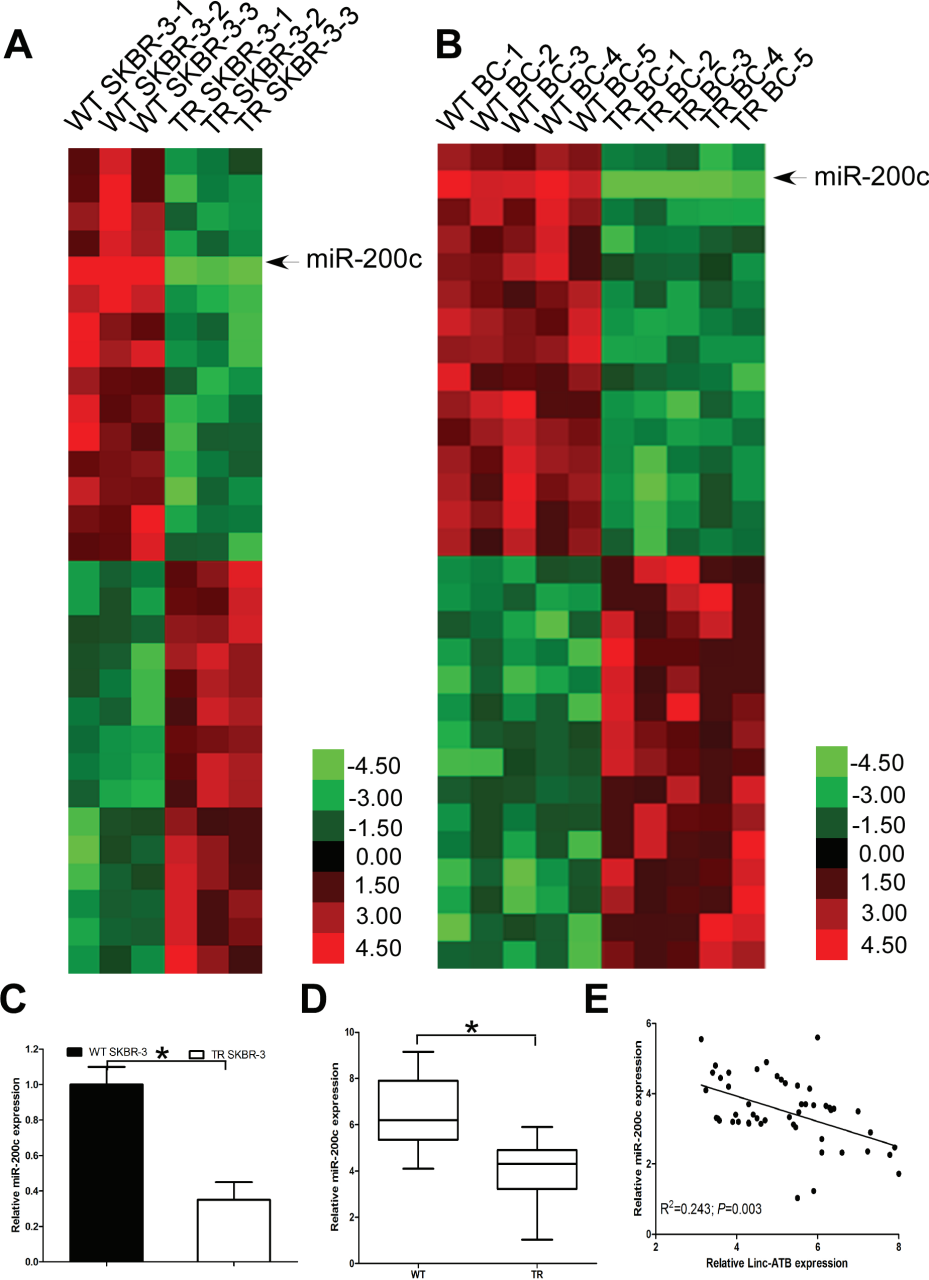
miR-200c is down-regulated and inverse correlated with lnc-ATB in TR breast cancer tissues and TR SKBR-3 cells **(A)** Expression profile of miRNAs in TR and WT SKBR-3 cells; **(B)** Expression profile of miRNAs in tissues of TR and WT breast cancer patients; **(C)** Expression of miR-200c in TR and WT SKBR-3 cells; **(D)** Expression of miR-200c in tissues of TR and WT breast cancer patients; **(E)** Relationship between miR-200c and lnc-ATB in tissues of 50 TR breast cancer patients. Data are expressed as means ± SD. Two-tailed Student's *t*-test was used to analyze the significant differences. *P < 0.05.

Previous studies reported lnc-ATB could function as a competing endogenous RNAs (ceRNA) by competitively biding common microRNAs [22], such as miR-200a, miR-200b, miR-200c, miR-141, and miR-429. More important, all these miRNAs had been reported to repress EMT and tumor invasion by targeting the 3′-UTR of ZEB1, ZEB2, and ZNF217, etc. Thus, we used microarray analysis to compare the miRNA expression level between the TR SKBR-3 and WT SKBR-3, we found 98 up-regulated and 73 down-regulated miRNAs in TR SKBR-3 cells. Then, we used microarray analysis to compare the miRNA expression level between the tissues of 5 WT breast cancer patients and 5 TR breast cancer patients, we found 54 up-regulated and 47 down-regulated miRNAs in TR breast cancer patients. Among the 30 miRNAs with the most significant fold change in tissues of TR breast cancer patients or TR SKBR-3 cells, miR-200c was found down-regulated in both TR SKBR-3 cells and tissues of TR breast cancer patients (Figure 4A and 4B), which is consistent with previous studies. We then further confirmed the significant down-regulation of miR-200c in TR SKBR-3 cells (Figure 4C) and tissues of 50 TR breast cancer patients (Figure 4D). Previous studies indicated lnc-ATB could function as a ceRNA by competitively binding miR-200c in hepatocellular carcinoma, so we investigated the relationship between lnc-ATB and miR-200c in breast cancer. As predicted, lnc-ATB inversely correlated with miR-200c expression in the tissues of TR breast cancer patients (Figure 4E).

### Lnc-ATB functions as a ceRNA by competitively biding miR-200c in TR SKBR-3 cells

**Figure 5:**
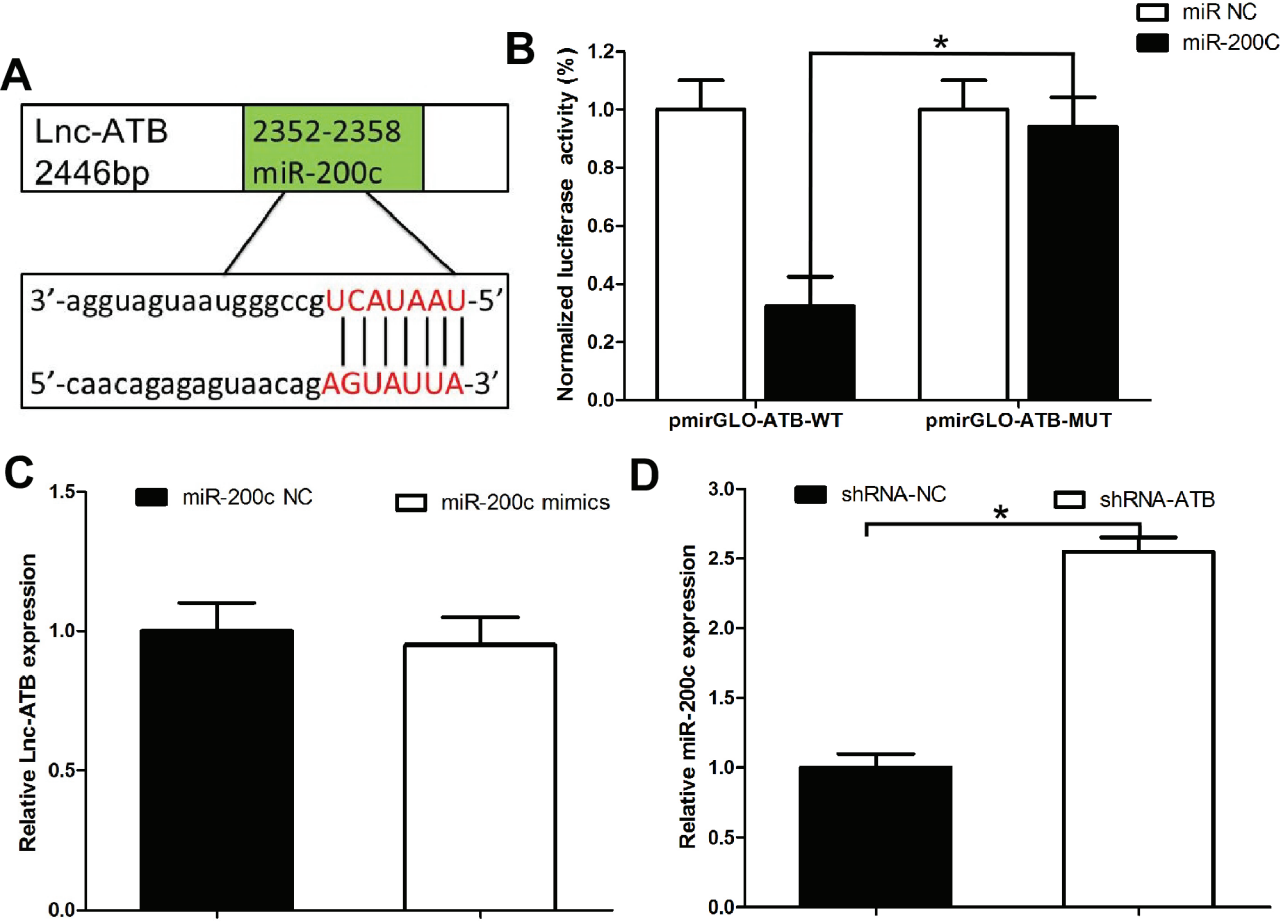
Lnc-ATB functions as a ceRNA by competitively biding miR-200c in TR SKBR-3 cells **(A)** Predicted binding sites of miR-200c on lncRNA-ATB; **(B)** Luciferase activity in 293T cells co-transfected with miR-200c and luciferase reporters containing lncRNA-ATB or mutant transcript. Data are presented as the relative ratio of firefly luciferase activity to renilla luciferase activity; **(C)** Expression of lnc-ATB in TR SKBR-3 cells transfected with miR-200c mimics and miR-200c control; **(D)** Expression of miR-200c in TR SKBR-3 cells transfected with lnc-ATB shRNA and shRNA control. Data are expressed as means ± SD. Two-tailed Student's *t*-test was used to analyze the significant differences. *P < 0.05.

To validate the direct binding between miR-200c and lnc-ATB at endogenous levels, we constructed luciferase reporters containing 3′ 500nt of lnc-ATB, which contains wild type (WT) or mutant (MUT) miR-200c binding sites (Figure 5A). We found overexpression of miR-200c reduced luciferase activities of WT reporter vector but not mutant reporter vector (Figure 5B). To determine whether lnc-ATB was regulated by miR-200c, we compared lnc-ATB expression in TR SKBR-3 cells transfected with miR-200c mimics or miR-200c control. We found overexpression of miR-200c did not induced the degradation of lnc-ATB in TR SKBR-3 cells (Figure 5C), which supporting that miR-200c was bona fide lnc-ATB-targeting miRNAs. But when the expression of lnc-ATB was down-regulated, the expression of miR-200c was corresponding up-regulated in TR SKBR-3 cells (Figure 5D). These results implied that lnc-ATB physically associated with miR-200c and functioned as a ceRNA for miR-200c in TR SKBR-3 cells.

### Lnc-ATB up-regulates and positive correlates with ZEB1 and ZNF217 levels

**Figure 6:**
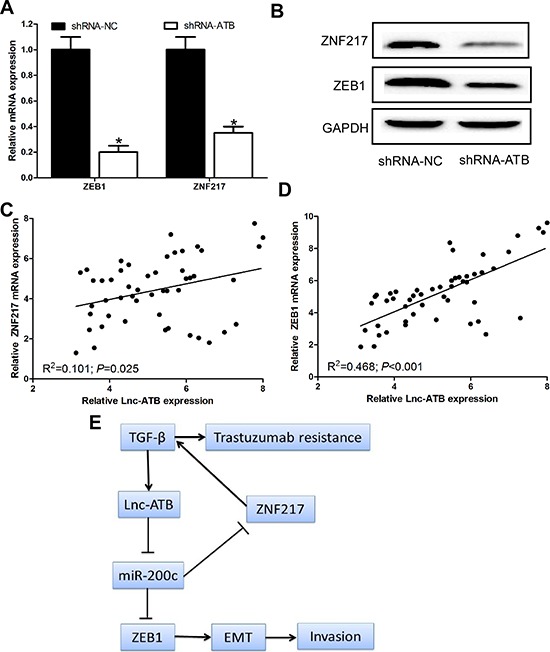
Lnc-ATB up-regulates and positive correlates with ZEB1 and ZNF217 levels **(A)** and **(B)** Protein and mRNA expression of ZNF217 and ZEB1 in TR SKBR-3 cells transfected with lnc-ATB shRNA and shRNA control; **(C)** Relationship between ZNF217 and lnc-ATB in tissues of 50 TR breast cancer patients; **(D)** Relationship between ZEB1 and lnc-ATB in tissues of 50 TR breast cancer patients; **(E)** A schematic model of lncRNA-ATB functions during the trastuzumab resistance and invasion-metastasis cascade. Data are expressed as means ± SD. Two-tailed Student's *t*-test was used to analyze the significant differences. *P < 0.05.

Because lnc-ATB shares regulatory miR-200c with ZEB1 and ZNF 217 which has been proven to be the direct targets of miR-200c in TR SKBR-3 cells, we wondered whether lnc-ATB could modulate ZEB1 and ZNF217. We found down-regulation of lnc-ATB could decrease the expression of ZEB1 and ZNF217 at mRNA and protein levels in TR SKBR-3 cells (Figure 6A and 6B). Additionally, lnc-ATB significantly correlated with the expression of ZNF217 and ZEB1 in the tissues of TR breast cancer patients (*P* = 0.025 and *P* < 0.001 respectively; Figure 6C and 6D).

## DISCUSSION

In the current study, we reported that lnc-ATB, which had been reported to be activated by TGF-β, promote trastuzumab resistance and invasion-metastasis cascade in breast cancer by competitively biding miR-200c, up-regulating ZEB1 and ZNF-217, and then inducing EMT (Figure 6E). In addition, we also found that the high level of lnc-ATB was correlated with trastuzumab resistance of breast cancer patients. All these data support our conclusion that lnc-ATB has pleiotropic effects on breast cancer cell invasion, apoptosis, and trastuzumab resistance.

Previous studies demonstrated the trastuzumab displayed potent inhibition of multi-organ metastasis [27], and loss of HER2 facilitated distal metastasis of breast cancer patients receiving trastuzumab treatment [28], which indicating that trastuzumab resistance significantly associated with metastasis in breast cancer. Furthermore, consistent with previous studies, our study also confirmed acquisition of trastuzumab resistance was characterized by enhanced invasiveness of breast cancer cells with concomitant EMT and elevated TGF-β signaling [26]. It has been demonstrated that lnc-ATB could mediate the role of TGF-β in induced EMT and promoting metastasis in hepatocellular carcinoma, colorectal cancer and breast cancer, which indicating lnc-ATB was a general TGF-β mediator. Furthermore, lnc-ATB was also proved to be a direct target of TGF-β/Smad pathway [22]. Our results indicated a higher level of lnc-ATB was associated with trastuzumab resistance in patients with breast cancer, which is consistent with the observation that elevated TGF-β signaling is also associated with trastuzumab resistance. More importantly, we also found miR-200c was down-regulated in the tissues of trastuzumab resistant breast cancer patients, and inversely correlated with lnc-ATB expression. It has been confirmed down-regulation of miR-200c promote invasiveness and EMT of breast cancer by regulating the expression of ZEB1 and ZNF217. Taken together our results and previous studies, we get the conclusion that both lnc-ATB and miR-200c play important role in the process of EMT and the malignant phenotype of TR SKBR-3 cells. Interestingly, it has been confirmed that lnc-ATB was a ceRNA by competitively binding miR-200c. Our study further confirmed lnc-ATB could regulate the expression of miR-200c, but miR-200c could not induce degradation of lnc-ATB. Thus, our results further elucidate lnc-ATB physically associate with miR-200c and function as a ceRNA in breast cancer cells. Considering the established role of miR-200c in EMT and trastuzumab resistance in breast cancer, we could conclude lnc-ATB which is activated by TGF-β modulate the process of trastuzumab resisitance and metastasis in HER2 positve breast cancer by regulating the miR200c/ZEB1 and miR-200c/ZNF217 signaling pathway.

In order to further confirm our hypothesis, we investigated the relationship between lnc-ATB and ZEB1/ZNF217. We found lnc-ATB shared miR-200c response elements with ZEB1 which was the master inducers of EMT and ZNF217 which promoted TGF-β by transcriptionally activating TGF-β2 and TGF-β3 [29–30]. We observed down-regulated lnc-ATB was sufficient to reduce the expression of ZEB1 and ZNF217 in TR SKBR-3 cells. Notably, this role depends on the competitive binding with miR-200c, further indicating lnc-ATB functions as a ceRNA in breast cancer cells. Because there is a double negative feedback loop between miR-200c and ZEB1/ZNF217 [31], the upregulation of ZEB1 and ZNF217 by lnc-ATB could further augment the effects. More importantly, the expression of lnc-ATB was significantly associated with ZEB1 and ZNF217 expression in TR breast cancer patients. All these results proved lnc-ATB functions as a ceRNA by regulating ZEB1 and ZNF217 expression in breast cancer.

ZNF 217 is a candidate oncogene in breast cancer, and overexpression of ZNF217 was found to promote migration and invasion of breast cancer cells *in vitro* and stimulate the development of spontaneous lung or lymph node metastasis *in vivo*. More importantly, ZNF was confirmed to promote EMT and mediate direct up-regulation of TGF-β2 and TGF-β3 in autocrine loop [29]. HER2 converts TGF-β from a tumor suppressor to a tumor promoter, and exogenous as well as transduced TGF-β confers motility and invasiveness to MCF10A nontransformed human mammary epithelial cells stably expressing transfected HER2 [32–33]. And GDF15-mediated activation of TGF beta receptor-Src-HER2 signaling crosstalk was reported to be a novel mechanism of trastuzumab resistance [34]. Taken together previous studies and our results, we tend to confirm lnc-ATB could augment its effects on trastuzumab resistance in breast cancer by lnc-ATB/ZNF217/TGF-β positive feedback. Thus, it is more prone to develop trastuzumab resistance in the breast cancer patients whose lnc-ATB expression was elevated.

More and more miRNAs have been proven to be associated with trastuzumab resistance in breast cancer. Specifically, miR-21 and miR-200c were confirmed to mediate trastuzumab resistance in breast cancer [25–26]. More importantly, nested feedback circuits of miR-200c/ZEB1 and miR-200c/ZNF217/TGF-β/ZEB1 contribute to EMT correlated with trastuzumab resistance and metastasis of breast cancers. Our studies expanded this regulatory circuit and identified an important role of lnc-ATB in this circuit. On one hand, the negative feedback between ZEB1 and miR-200c may be more complicated than we currently know. Lnc-ATB may be function as a ceRNA to up-regulate the ZEB1 expression in breast cancer. On the other hand, lnc-ATB could enlarge its effect on trastuzumab resistance and EMT of breast cancer by lnc-ATB/ZNF217/TGF-β signaling pathway. Thus, up-regulation of miR-200c alone may not sufficient to reverse trastuzumab resistance in breast cancer. Therefore, nested feedback circuits of lnc-ATB/miR-200c/ZEB1 and lnc-ATB/miR-200c/ZNF217/TGF-β/ZEB1 contribute to EMT correlated with trastuzumab resistance and metastasis of breast cancer.

Taken together, our research demonstrated that lnc-ATB acts as a key regulator of TGF-β signaling pathways and restuzumab resistance in breast cancer. The findings of this study have significant implications regarding our understanding the mechanism of EMT and trastuzumab resistance in breast cancer. As direct targets of lnc-ATB, miR-200c-ZEB1/ZNF217 mediated the role of lnc-ATB in trastuzumab resistance and invasion-metastasis cascade. The pleiotropic effects of lnc-ATB on the trastuzumab resistance and invasion-metastasis cascade suggest that lnc-ATB could be an effective target for anti-metastasis and reversal of trastuzumab resistance therapies.

## METHOD AND MATERIALS

### Cell culture

Human breast cancer SKBR-3 cell line was purchased from the Cell Bank of Chinese Academy of Sciences (Shanghai, China), where they were characterized by mycoplasma detection, DNA-Fingerprinting, isozyme detection and cell vitality detection. Cells were cultured in RPMI 1640 medium (Invitrogen, Carlsbad, CA) supplemented with 15% fetal bovine serum (FBS, Hyclone, Logan, UT) and maintained at 37°C in a humidified incubator with 5% CO2. Recombinant TGF-β2 (BioLegend, San Diego, CA) and Docetaxel (Sigma-Aldrich, St. Louis) were used for incubation with cells at working concentrations of 20 ng/ml and 4 nM, respectively. Trastuzumab (Herceptin) was obtained from Roche (Basel, Switzerland) and dissolved in sterile water. Trastuzumab-resistant (TR) cells were developed by continuous culture of SKBR-3 cells in the presence of 5 mg/ml trastuzumab for 6 months as reported.17 Thereafter, TR and parental (WT) SKBR-3 cells were cultured in parallel with or without trastuzumab, respectively.

### Microarray analysis

The total RNA was extracted from above mentioned TR or WT SKBR-3 cells and breast cancer patients, amplified and transcribed into fluorescent cRNA using the Quick Amp Labeling kit (Agilent Technologies, Palo Alto, CA). The labeled cRNA was then hybridized onto the Human LncRNA Array v2.0 (8 × 60K, ArrayStar, Rockville, MD), and after the washing steps, the arrays were scanned by the Agilent Scanner G2505B. Agilent Feature Extraction software (version 10.7.3.1) was used to analyze acquired array images. Quantile normalization and subsequent data processing were performed using the GeneSpring GX v11.5.1 software package (Agilent Technologies). The differentially expressed lncRNAs and miRNAs with statistical significance were identified using volcano plot filtering. The thresholds we used to screen up-regulated or down-regulated lncRNAs and miRNAs are fold change > = 1.5 and a *p*-value < = 0.05. Gene expression profiles were determined using Phalanx human OneArray microarrays (HOA 6.1) following the manufacturer's instructions.

### MTT assay

Cell proliferation was analyzed *in vitro* with the tetrazolium salt 3-(4, 5-dimethylthiazol-2-yl)-2, 5-diphenyltetrazolium bromide (MTT) reagent. Briefly, 2000 cells from each group were plated in each well of five 96-well plates in 200 μL of medium. To analyze cell proliferation, 20 μL of MTT substrate at a concentration of 2.5 mg/mL in PBS was added to each well. The plates were then returned to a standard tissue incubator for an additional 4 h. The medium was then removed, and the cells were solubilized in 150 μL of dimethylsulfoxide for the colorimetric analysis (wavelength, 490 nm). One plate was analyzed immediately after the cells adhered (approximately 4 h after plating). Then, one plate per day was examined for the next 4 consecutive days.

### Apoptosis assay

TR SKBR-3 cells were transfected with lnc-ATB shRNA and shRNA control for 48 h, and the cells were then suspended in incubation buffer at a density of 1 × 106 cells/mL. The cells were incubated with Annexin V-FITC and propidium iodide (PI) (BD Bioscience, San Jose, CA) for 15 min in the dark at room temperature. Cell apoptosis was then analyzed using FACSCalibur (BD Biosciences, San Diego, CA, USA).

### Migration and invasion assay

Cell migration and invasion capacity were measured using transwell migration assays (Millipore, Billerica, MA) *in vitro*. The TR SKBR-3 cells were transfected with lnc-ATB shRNA and shRNA control for 48 h, and then the cells were suspended in RPMI-1640 with 10 g/L BSA at a density of 1×106 cells/mL. Then, cell suspensions (150 μL) were seeded in the upper chamber with aporous membrane coated with (for the transwell invasion assay) or without (for the migration assay) Matrigel (BD Bioscience, San Diego, CA). To attract the cells, 500 μL of RPMI-1640 with 10% serum was added to the bottom chamber. After allowing the cells to migrate for 24 h or to invade for 48 h, the penetrated cells on the filters were fixed in dried methanol and stained in 4 g/L crystal violet. The numbers of migrated or invasive cells were determined from five random fields using a microscope (Olympus) at × 10 magnification.

### Quantitative RT-PCR for miRNAs and mRNAs

Total RNA was extracted by Trizol reagent (Invitrogen, Carlsbad, CA) according to the manufacturer's protocol. For mRNA, reverse transcription reaction was performed with SuperScriptTM II reverse transcriptase (Invitrogen, Carlsbad, CA). cDNA was detected using SYBR@ Premix Ex TaqTM (TaKaRa Bio Group, Shiga, Japan). For quantifying miRNAs, a miScript reverse transcription kit (Qiagen, Hilden, Germany) was used for reverse transcription, followed by amplification using SYBR@ Premix Ex TaqTM (Takara). GAPDH and U6 RNA were used as internal loading controls for mRNAs and miRNAs, respectively. The detailed information of primers for qPCR are listed in Supplementary Table 1.

### Western blot analysis

Cells were washed in phosphate buffered saline (PBS) twice before proteins were extracted, and proteins were separated on a SDS/PAGE gel, transferred onto a PVDF membrane and subjected to immunoblot analysis. Blotting was performed with antibodies against E-cadherin (ab53226, Abcam, Cambridge, UK), vementin (ab8978, Abcam, Cambridge, UK), ZEB1 (ab180905, Abcam, Cambridge, UK), ZNF217 (ab136678, Abcam, Cambridge, UK). Goat anti-rabbit and goat anti-mouse immunoglobulin horseradish peroxidase-linked F(ab)2 fragments (ZAGB-bio, Beijing, China) were used as secondary antibodies.

### Luciferase reporter assay

pmirGLO-ATB-WT or pmirGLO-ATB-MUT(miR-200c) was coransfected with miR-200c mimics or miR NC into 293T cells by Lipofectamine-mediated gene transfer. The relative luciferase activity was normalized to Renilla luciferase activity 48 hr after transfection.

### Statistical analysis

Statistical analysis was performed using IBM SPSS statistical software (version 21.0). The differences in characteristics between the 2 groups were examined by the χ2 test and Fisher's exact test. All *P*-values were determined from 2-sided tests, and statistical significance was based on a *P*-value of 0.05.

### Patients and specimens

This study was approved by the Research Ethics Committee of No.202 of PLA. Written informed consent was obtained from all of the patients. All specimens were handled and made anonymous according to the ethical and legal standards. 50 cases of herceptin-sensitive and herceptin-resistant breast cancer patients based on accepted clinicopathological were enrolled in this study. The inclusion criteria of herceptin-resistant breast cancer patients were as following: 1, primary herceptin-resistance: the condition of patients who recieved initial trastuzumab treatment (12 weeks or less) progressed; 2, secondary herceptin-resistance: the condition of patients who reveived innitial trastuzumab treatment was under control, but the disease was progressed during the treatment (>12 weeks).

## SUPPLEMENTARY TABLE



## Abbreviations

TR: Trastuzumab resistant
miRNAs: microRNAs
qRT-PCR: Quantitative Real-time Polymerase Chain Reaction
lnc-ATB: Long noncoding RNA Activated by TGF-β
3′-UTR: 3′-untranslated region
MTT: 3-(4, 5-dimethylthiazol-2-yl)-2, 5-diphenyltetrazolium bromide
MUT: Mutant
WT: Wild type
PI: Propidium iodide
HER2: Human epidermal growth factor receptor 2
EMT: Epithelial-mesenchymal transition
