# Supplementary material for: LncRNA-ATB promotes trastuzumab resistance and invasion-metastasis cascade in breast cancer
Source: Oncotarget. 2015 Mar 23;6(13):11652–63. doi: 10.18632/oncotarget.3457 (PMC4484483; doi:10.18632/oncotarget.3457)
Supplement: Supplementary file 1 [file oncotarget-06-11652-s001.pdf]

SUPPLEMENTARY TABLE

Supplementary Table 1: Primers used for qRT-PCR

Gene name	Forward primer 5′-3′	Reverse primer 5′-3′
ZEB1	GACAGTGTTACCAGGGAGGAGCA	TTCAGGTGCCTCAGGAAAAATGA
ZNF217	CTGAAACGGGGAAGAAGCCT	CTTGCCCCGATTCCTTCACT
ZEB2	GGTATTGCCAACCCTCTG	CTCCCTTATTTCATCTTCCTCT
Snail1	ACAAGCACCAAGAGTCCG	ATGGCAGTGAGAAGGATGTG
ETS1	ACAGGGTAAGTGAAGGTTAATTCCA	AGAAAGATGACTACCTTGCTTGACT
Twist1	AGCTGAGCAAGATTCAGACCC	GCAGCTTGCCATCTTGGAGT
